# Therapeutic effect of high‐frequency ultrasound‐assisted dye laser on hemangioma and its influence on serum HIF‐1α in patients

**DOI:** 10.1002/jcla.22970

**Published:** 2019-09-30

**Authors:** Hongzhang Hu, Pengyuan Song, Jinyan Yang, Xia Wang, Zhaohui Chen, Jianhua Fang

**Affiliations:** ^1^ Department of Laboratory Medicine, West China Hospital Sichuan University Chengdu China; ^2^ Department of Ultrasound The Second Affiliated Hospital of Xinjiang Medical University Urumqi China; ^3^ Department of Dermatology The Second Affiliated Hospital of Xinjiang Medical University Urumqi China; ^4^ Department of Ultrasonography, Affiliated Hangzhou First People’s Hospital Zhejiang University School of Medicine Hangzhou China

**Keywords:** hemangioma, HFU, HIF‐1α, laser

## Abstract

**Background:**

To analyze the therapeutic effect of high‐frequency ultrasound (HFU)‐assisted dye laser on hemangioma patients and changes in serum hypoxia‐inducible factor‐1α (HIF‐1α).

**Methods:**

A total of 20 patients diagnosed with hemangioma in our hospital from January 2013 to March 2018 were selected, including 12 males and eight females. All patients were treated with HFU‐assisted dye laser. The site and type of hemangioma and age distribution of patients were collected, and changes in data and area of hemangioma and serum HIF‐1α before and after treatment were analyzed.

**Results:**

The vascular condition of hemangioma in all patients was significantly improved at 7, 14, and 30 days after treatment. Gray‐scale ultrasound displayed that the tumor area was reduced by more than 50%. After treatment, the serum HIF‐1α level declined obviously after treatment compared with that before treatment, showing a statistically significant difference (*P* < 0.05).

**Conclusion:**

HFU‐assisted dye laser can effectively reduce the tumor area, decrease the serum HIF‐1α level, and improve the prognosis in the treatment of hemangioma.

## 1 INTRODUCTION

Deformity of vascular morphology and hemangioma are common in vascular diseases in clinic, more than half of which are located in the head, neck, and face.^1^ Hemangioma is also a kind of common benign subcutaneous tumor, and its most common cause is the venous distention and malformation due to abnormal embryonic development, which is generally manifested as invasive growth and medium or soft texture.^2^ The local shape and size of hemangioma can be observed by naked eyes, and the concrete depth and range of tumor invasion can be detected via such imaging methods as high‐frequency ultrasound (HFU) and angiography.^3^ Common therapeutic methods for hemangioma include injection of hardening agent, local injection of hormone, chemoradiotherapy, and vascular embolization.^4^ LASERs (light amplification by stimulated emission of radiation) represent sources of high‐intensity monochromatic (single wavelength) coherent light that can be used for the treatment of various dermatologic conditions depending on the wavelength, pulse characteristics, and fluence (energy output). A dye laser employs an organic dye mixed in a solvent as the lasing medium. The dyes include rhodamine, fluorescein, coumarin, stilbene, umbelliferone, tetracene, and malachite green while the solvents used contain water, glycol, ethanol, methanol, hexane, cyclohexane, and cyclodextrin. The dye solution is usually circulated at high speeds, to promote the dye molecules into the state of being ready to emit stimulated radiation. Pulsed dye lasers produce pulses of visible light at a wavelength of 585 or 595 nm with pulse durations of the order of 0.45‐40 ms, which can be combined with radiofrequency to enhance effects.^5^ With the constant development of laser medicine, HFU‐assisted dye laser has many advantages in the treatment of hemangioma, such as small trauma and low risk of scar after treatment.^6^ Hemangioma not only affects the appearance, but also leads to a variety of local or systemic complications, such as ulceration, bleeding, and even infection on local surface. Hemangioma located in special sites probably results in dyspnea and diminution of vision, thus threatening the life.^7^ The main principle of treatment of hemangioma with HFU‐assisted dye laser is the target chromophore. The target chromophore of hemangioma is the oxidized hemoglobin in blood vessels, and the laser can make oxyhemoglobin generate heat through absorbing light energy, thus causing vascular injury.^8^, ^9^ With the constant development of laser medical technology, HFU‐assisted dye laser has promoted the treatment of hemangioma.

## 2 GENERAL MATERIALS AND METHODS

### 2.1 General materials

A total of 20 patients diagnosed with hemangioma in our hospital from January 2013 to March 2018 were selected, including 12 males and 8 females aged 3 months to 35 years old with an average age of (17.05 ± 15.17) years old. The type and site of hemangioma and age distribution of all patients were recorded (Table 1). All patients enrolled were treated with 595 mm dye laser therapy in our hospital using the 595 mm pulsed dye laser therapeutic instrument provided by Medical Laser Company, USA. Conditions of patients were recorded before and after HFU therapy. Before treatment, all patients were informed of the mechanism, main efficacy, and possible side effects of HFU‐assisted dye laser (pain during treatment, redness, swelling, and itching immediately after the procedure that may last a few days after treatment, blistering, changes in skin pigmentation, bruising a bacterial infection) and asked to be reviewed within the stipulated time after treatment. Before treatment, informed consent was obtained from patients and their families, and related files were signed.

**Table 1:** Cases of different sites and types of hemangioma and age distribution

Site	Type of hemangioma			Age distribution (years old)			
	Nevus flammeus	Strawberry	Spongy	0‐3	3‐10	10‐25	>25
Forehead		/					/
Upper eyelid	/			/			
Lower eyelid			/		/		
Tip of nose		/		/			
Near left nose		/				/	
Right cheek	/				/		
Upper lip			/				/
Lower lip			/			/	
In front of ear		/			/		
Behind ear		/			/		
Below chin	/			/			
Middle neck			/				/
Root of neck			/			/	
Upper abdomen			/	/			
Waist	/				/		
Forehead		/				/	
Middle neck	/				/		
Hip	/					/	
Thenar		/		/			
Root of middle finger	/					/	

### 2.2 Methods

Therapeutic method: Relevant data of patients were collected before treatment, including age, gender, and site, size, scope, color, and type of hemangioma. The hemangioma was photographed using the same camera from the same angle, light, and position and archived by the same professional physician. The skin of patients was cut to fully expose the tumor, and the surface morphology, boundary, scope, size, invasion depth, texture, and blood flow distribution in internal vessels of hemangioma were observed using the direct detection method. First, after routine surface cleaning, the local skin was disinfected, and anesthesia was not performed. Before treatment, an appropriate amount of lidocaine was applied on the focus according to the patient's tolerance and local surface area of hemangioma, and the lidded transparent plastic magnet was pasted onto the focus for about 1 hour, followed by ultrasonic therapy. Before treatment, patients were fully informed of the related risk and curative effect. During treatment, the laser handle was always perpendicular to the focus to ensure the laser radiation to the whole treatment region, the overlapping region of light spot was approximately 10%, and the appropriate energy density was selected based on the color, size, and depth of blood vessels in hemangioma. Basically, 595 nm Venous flashlamp pulsed dye laser at a pulse duration of 0.45‐40 ms, spot diameter of 4‐7 mm, and energy fluence of 6.0‐12.5 J/cm^2^ was performed. The parameters of laser therapy might be slightly adjusted according to the patient's age and type, site, and color of hemangioma. The local color changing from dark to light after radiation indicated the appropriate energy density. In case that hemangioma showed the dotted structure and could be fully overlapped, and hemangioma was located around the eye or in the eyelid, patients were asked to wear the eye shield before treatment to protect the eyes. After treatment, erythromycin ointment was applied locally, followed by sun protection, cleaning, etc. At 1 week, 2 weeks, and 1 month after treatment, patients were reviewed, and HFU detection was performed for changes in the area of hemangioma, the number of blood vessels in hemangioma, peak flow velocity (*V*_max_) of main artery, and changes in the blood resistance index (RI).

### 2.3 Statistical methods

Statistical Product and Service Solutions (SPSS) 19.0 software was used for data processing. Data were collected and expressed as ($\bar{\chi }\bar{\chi }$ ± s), and *t* test was adopted for the sample mean. *P* < 0.05 suggested that the difference was statistically significant.

## 3 RESULTS

### 3.1 Main data of hemangioma detected via HFU before treatment

In hemangioma in different sites before treatment, there were abundant blood flow signals, a large number of blood vessels, and higher *V*_max_ of main artery (Table 2).

**Table 2:** Main data of hemangioma detected via HFU before treatment

Site	Blood flow signal	Number of blood vessels (n)	*V*_max_ of main artery (m/s)	RI of main artery
Forehead	Dendritic and lobulated shape	6	0.35	0.31
Upper eyelid	Arteriovenous communication	3	0.58	0.30
Lower eyelid	Single passing	2	0.47	0.30
Tip of nose	Single passing	1	0.40	0.45
Near left nose	Arteriovenous communication	3	0.35	0.53
Right cheek	Dendritic and lobulated shape	4	0.35	0.40
Upper lip	Arteriovenous non‐communication	4	0.55	0.37
Lower lip	Arteriovenous communication	3	0.44	0.42
In front of ear	Dendritic and lobulated shape	2	0.29	0.35
Behind ear	Arteriovenous non‐communication	2	0.33	0.32
Below chin	Arteriovenous communication	4	0.26	0.31
Middle neck	Dendritic and lobulated shape	5	0.25	0.47
Root of neck	Arteriovenous communication	4	0.27	0.42
Upper abdomen	Dendritic and lobulated shape	4	0.38	0.53
Waist	Arteriovenous communication	2	0.42	0.51
Hip	Arteriovenous non‐communication	4	0.37	0.47
Thenar	Arteriovenous non‐communication	1	0.40	0.45
Root of middle finger	Arteriovenous non‐communication	2	0.38	0.40

### 3.2 Main data of hemangioma detected via HFU at 7 days after treatment

In hemangioma in different sites at 7 days after treatment, blood flow signals weakened, and the number of blood vessels and *V*_max_ of main artery declined compared with those before treatment (Table 3).

**Table 3:** Main data of hemangioma detected via HFU at 7 d after treatment

Site	Blood flow signal	Number of blood vessels (n)	*V*_max_ of main artery (m/s)	RI of main artery
Forehead	Dendritic and lobulated shape	3	0.23	0.45
Upper eyelid	Arteriovenous communication	1	0.24	0.52
Lower eyelid	Single passing	1	0.23	0.38
Tip of nose	Single passing	1	0.21	0.29
Near left nose	Arteriovenous communication	1	0.30	0.45
Right cheek	Dendritic and lobulated shape	2	0.27	0.42
Upper lip	Arteriovenous non‐communication	1	0.33	0.41
Lower lip	Arteriovenous communication	1	0.31	0.47
In front of ear	Dendritic and lobulated shape	1	0.25	0.37
Behind ear	Arteriovenous non‐communication	1	0.24	0.38
Below chin	Arteriovenous communication	2	0.19	0.35
Middle neck	Dendritic and lobulated shape	2	0.20	0.32
Root of neck	Arteriovenous communication	1	0.21	0.37
Upper abdomen	Dendritic and lobulated shape	2	0.25	0.55
Waist	Arteriovenous communication	1	0.27	0.47
Hip	Arteriovenous non‐communication	2	0.29	0.32
Thenar	Arteriovenous non‐communication	1	0.25	0.35
Root of middle finger	Arteriovenous non‐communication	1	0.30	0.30

### 3.3 Main data of hemangioma detected via HFU at 14 days after treatment

In hemangioma in different sites at 14 days after treatment, there were basically no or very weak blood flow signals, and the number of blood vessels and *V*_max_ of main artery obviously declined (Table 4).

**Table 4:** Main data of hemangioma detected via HFU at 14 d after treatment

Site	Blood flow signal	Number of blood vessels (n)	*V*_max_ of main artery (m/s)	RI of main artery
Forehead	Dendritic and lobulated shape	1	0.21	0.28
Upper eyelid	Arteriovenous communication	0	/	/
Lower eyelid	Single passing	0	/	/
Tip of nose	Single passing	1	0.17	0.20
Near left nose	Arteriovenous communication	0	0.19	0.30
Right cheek	Dendritic and lobulated shape	1	/	/
Upper lip	Arteriovenous non‐communication	0	0.21	/
Lower lip	Arteriovenous communication	0	/	/
In front of ear	Dendritic and lobulated shape	1	0.18	0.29
Behind ear	Arteriovenous non‐communication	0	/	/
Below chin	Arteriovenous communication	1	/	/
Middle neck	Dendritic and lobulated shape	0	0.17	0.28
Root of neck	Arteriovenous communication	1	/	0.29
Upper abdomen	Dendritic and lobulated shape	0	0.20	0.30
Waist	Arteriovenous communication	0	0.15	/
Hip	Arteriovenous non‐communication	1	0.23	/
Thenar	Arteriovenous non‐communication	0	0.20	0.30
Root of middle finger	Arteriovenous non‐communication	0	0.18	0.27

### 3.4 Main data of hemangioma detected via HFU at 1 month after treatment

At 1 month after treatment, blood flow signals of hemangioma could be detected only in the forehead, upper abdomen, waist, and hip, while blood flow signals, blood vessels, and *V*_max_ of main artery were not detected in other sites (Table 5).

**Table 5:** Main data of hemangioma detected via HFU at 1 mo after treatment

Site	Blood flow signal	Number of blood vessels (n)	*V*_max_ of main artery (m/s)	RI of main artery
Forehead	Single passing	1	0.21	0.22
Upper abdomen	Dendritic and lobulated shape	1	0.20	0.28
Waist	Arteriovenous communication	0	/	/
Hip	Arteriovenous non‐communication	1	0.28	0.28
Others	/	/	/	/

### 3.5 Changes in the area of hemangioma under HFU after treatment

Gray‐scale ultrasound displayed that the area of hemangioma in different sites was reduced by more than 50%. Except 1 case of large‐area hemangioma below the chin that needed longer‐term follow‐up or would be treated with other means eventually, the recovery of hemangioma in other sites was significant (Table 6).

**Table 6:** Changes in the area of hemangioma under HFU after treatment (7, 14, and 30 d)

	7 d after treatment	14 d after treatment	30 d after treatment
Detection of changes in the area of hemangioma via HFU	1 case in forehead 1 case in front of ear The relatively strong‐echo area is reduced by 50% under HFU	1 case in forehead 1 case in front of ear 1 case in tip of nose 1 case in lower eyelid 1 case below chin The relatively strong‐echo area is reduced by 50%‐75% under HFU	Except 1 case below chin The echo area of hemangioma in other sites is reduced by 50%‐75% under HFU
	2 cases	3 cases	23 cases

### 3.6 Changes in the mean percentage of decline in echo area of hemangioma under HFU after treatment

The mean percentages of decline in echo area of nevus telangiectaticus, capillary hemangioma, and cavernous hemangioma were significantly increased at 14 days after treatment compared with those at 7 days after treatment and also obviously increased at 1 month after treatment compared with those at 7 and 14 days after treatment, displaying statistically significant differences (*P* < 0.05) (Table 7).

**Table 7:** Changes in the mean percentage of decline in echo area of hemangioma under HFU after treatment

After treatment	Nevus telangiectaticus	Capillary hemangioma	Cavernous hemangioma
7 d	0.25 ± 0.05	0.31 ± 0.03	0.11 ± 0.03
14 d	0.39 ± 0.12	0.42 ± 0.06	0.19 ± 0.04
30 d	0.78 ± 0.08	0.81 ± 0.11	0.27 ± 0.05

### 3.7 Changes in serum HIF‐1α before and after treatment

The level of serum HIF‐1α was remarkably decreased after treatment compared with that before treatment, and it was lower at 14 days after treatment than that at 7 days after treatment and also lower at 1 month after treatment than that at 7 and 14 days after treatment, showing statistically significant differences (*P* < 0.05) (Table 8).

**Table 8:** Changes in serum HIF‐1α before and after treatment

Time	HIF‐1α (pg/mL)
Before treatment	253.07 ± 41.22
7 d after treatment	182.77 ± 50.73
14 d after treatment	156.23 ± 40.86
30 d after treatment	109.86 ± 30.72

## 4 DISCUSSION

Hemangioma is a kind of vascular disease of congenital or acquired developmental anomaly of blood vessels, which is generally caused by abnormal proliferation of vascular endothelial cells and pericytes.^10^ Hemangioma can occur in any part of the body in the continuous development of disease, emerge in a kind of tissue, or invade different layers of tissues, leading to local or systemic complications.^11^ It mainly occurs in skin mucosa or oral mucosa, and cavernous hemangioma and capillary hemangioma are dominated.^12^ Cavernous hemangioma is composed of significantly enlarged vascular lumen and cavity sinus, the latter of which has a single layer of endothelial cells, and vascular lumen and vascular sinus are separated by connective tissues.^13^ The structure and nature of cavernous hemangioma are similar to those of sponge, and it is rich in venous blood.^14^ X‐ray displays that the arteries and branches in hemangioma in the arterial phase are generally normal, hemangioma cannot be developed, but hemangioma in the venous phase can be developed, further confirming that hemangioma is derived from the vein.^15^ In the past, common therapeutic methods for hemangioma included local skin grafting, isotope therapy, local injection of hormone, and local electrocoagulation,^5^ which was less targeted, and had a therapeutic scope far beyond the focus, large trauma, and stronger inflammatory response later.^16^ In the late repair of these therapeutic methods, fibrous protein is often formed, resulting in a high risk of scar formation.^17^ With the further development of ultrasonic laser technique, HFU‐assisted dye laser, as a special targeted intensive treatment, has offered more possibilities for the universal aesthetic requirements.^18^ HFU laser therapy is strongly targeted at oxyhemoglobin in the blood, the probability of occurrence of inflammatory response is low after treatment, and it causes very little damage to deep tissues with a low risk of scar formation after treatment.^18^ Out data indicated that, in different sites of hemangioma, the blood flow signals as well as the number of blood vessels and *V*_max_ of main artery gradually declined. At 1 month after treatment, blood flow signals of hemangioma could be detected only in the forehead, upper abdomen, waist, and hip, while blood flow signals, blood vessels, and *V*_max_ of main artery were not detected in other sites. We propose that the efficacy of 595 mm dye laser therapy may depend on the severity of the disease while the actual efficacy is likely to have certain correlation with the site of hemangioma according to a retrospective study indicating that the early treatment of superficial hemangioma contributes to excellent clearance rate and only few adverse effects.^19^ In this study, it was found that the blood flow signals, the number of blood vessels and the area of hemangioma were dramatically decreased after treatment. However, there were also some adverse reactions in the treatment of hemangioma with HFU‐assisted dye laser. In this study, there were 2 cases of allergic reaction, 1 case of pigment loss and 1 case of skin tightening after treatment with HFU‐assisted dye laser, and the injury could be effectively reduced by appropriate energy and pulse width. Serum HIF‐1α is a regulatory factor with a decisive effect in hypoxic tumor cells. The severer the environmental hypoxia is, the more stable HIF‐1α will be, thereby helping enhance stability and proliferation capacity of tumor cells.^19^ Some studies have demonstrated that the stronger the expression of nuclear antigen in tumor cells is, the higher the level of HIF‐1α will be, the synthesis and secretion of HIF‐1α are promoted by tumor cells due to hypoxia, and the level is closely related to angiogenesis.^20^, ^21^

## 5 CONCLUSION

Results of this study revealed that the level of serum HIF‐1α sharply declined after treatment of hemangioma with HFU‐assisted dye laser, confirming the treatment effect of ultrasonic laser therapy on hemangioma.
